# Supplementary material for: Single vs replicate Real-Time PCR SARS-CoV-2 testing: Lessons learned for effective pandemic management
Source: PLoS One. 2022 Jul 14;17(7):e0269883. doi: 10.1371/journal.pone.0269883 (PMC9282535; doi:10.1371/journal.pone.0269883)
Supplement: Attachment — Submitted filename: Response to reviewers.docx [file pone.0269883.s002.docx]

Dear Editor,

Please find our revised manuscript. We thank the three reviewers for their comments. We have revised the manuscript accordingly and provide specific answers below.

Reviewer #1: 
In this report, the authors compare the accuracy estimates of two SARS-CoV-2 testing protocols, Vitassay One-Step Real-Time RT-PCR and Primerdesign One-Step Real-Time RT-PCR assay, specifically showing the need for replicate testing considering their accuracies. While the paper presents results that are scientifically sound and reiterate previously published accuracy estimates of SARS-CoV-2 tests, it does not address a sufficiently original research question to merit publication in PLoS One. Most diagnostic tests have non-zero error rates which means accuracy will be better with replicate testing. The paper doesn’t add novel value as to situations in the ongoing COVID-19 pandemic where replicate testing will be specifically necessary, e.g., certain variants, populations, or viral loads.

We thank the reviewer for the comments and review of our work. We believe that the originality of our research question and work lies on the investigation of a significantly large cohort of clinical samples and the accurate calculation of the RT-PCR detection assay based error in singlet vs doublet testing.

Our findings demonstrate that a singlet testing policy would have failed to detect approximately 19% of SARS-CoV-2 positive patients, approximately 94 in 10,000 patients and a predictive ratio of 940/100,000 individuals. This level of false negatives, in correlation with an R_0_ number >1, can contribute to sustained and increasing SARS-CoV-2 infection rates within hospitals, healthcare settings and the wider community.

We believe that this observation is significant and important in line of the increased transmissibility of SARS-CoV-2 variants including the Omicron variant and the likelihood that significantly altered immunity evading variants may arise in the future.

While it is true that, as mentioned by the reviewer, most diagnostic tests have non-zero error rates our work addresses the testing policy rather than the limitations of the different diagnostic kits.

In addition, while the error rate of SARS-CoV-2 diagnostic testing has been defined as a wide estimate, our study concludes a precise error value attributed to testing protocols only.

Although it is clear that testing decisions need to be made in times of emergency, such as this pandemic, we believe that our work can provide an accurate error rate that can be built into viral spread and pandemic modelling in order to provide real-life epidemiological models for the health services and policy makers. This is applicable to this as well as future pandemics, where similar testing protocols and policies can be employed.

Reviewer #2: 
Interesting confirmatory paper in real field on the high risk of false negative results for SARS-CoV-2 RNA detection in clinical samples. Two different PCR assays were used on high number of clinical samples. The paper is short and well written.

Minor points
P5. Give the periods of inclusion.
P5. Please indicate the percentages in paragraphs 3 and 4.
P8. Update the reference 7:
Arevalo-Rodriguez I, Buitrago-Garcia D, Simancas-Racines D, Zambrano-Achig P, Del Campo R, Ciapponi A, Sued O, Martinez-García L, Rutjes AW, Low N, Bossuyt PM, Perez-Molina JA, Zamora J. False-negative results of initial RT-PCR assays for COVID-19: A systematic review. PLoS One. 2020 Dec 10;15(12):e0242958.

We thank the reviewer for the comments and review of our work. We can confirm that all comments have been addressed in the manuscript.

P5: “A total of 10,014 nasopharyngeal swabs were tested using 20,028 duplicate Real-Time PCR reactions. The tested nasopharyngeal swabs were collected between the months of May and July 2020”.

P5: “Out of the 10,014 patients, 678 (corresponding to 1,356 duplicate Real-Time RT-PCR reactions) were tested with the Vitassay One-Step Real-Time RT-PCR kit. Of these, 27 patients (3.98%) were reported as positive, with 23 (85% of all positive patients) detected as duplicate positives (detection was observed in both replicates). In addition, 1 patient was detected as positive on replicate 1 only and 3 patients were detected as positive on replicate 2 only. Therefore, 4 patients were detected positive on one replicate (15% of all positive patients).

The Primerdesign One-Step Real-Time RT-PCR assay was used to test 9,336 patients (corresponding to 18,672 duplicate Real-Time RT-PCR reactions). Of these, a total of 464 patients (4.97%) were detected as SARS-CoV-2 positive, with 279 patients (60% of all positive patients) detected as duplicate positive (detection on replicate 1 and replicate 2), 84 patients detected positive on replicate 1 only and 101 patients detected as positive on replicate 2 only. Overall, 185 patients were detected positive on one replicate (40% of all positive patients)”.

P8: The reference has been updated.

Reviewer #3: 
Summary:
The authors us data from real-time PCR testing of 10,014 nasopharyngeal swab specimens to compare the rate of detection of SARS-CoV-2 in single vs replicate testing scenarios. The authors make the argument that replicate testing provides a superior detection mechanism for ensuring accurate test results for pandemic control. Of the original 10,014 swabs, 9336 were tested twice using the Vitassay One-Step Real-Time RT-PCR assay, which uses 2 SARS-CoV-2 specific primer/probe set; and 678 swabs were tested using the Primerdesign LTD, Coronavirus (COVID-19) Genesig Real-Time PCR assay, which uses 1 SARS-CoV-2 specific primer/probe set. The authors report that, in aggregate, 2.45% of samples tested positive on either the first, second, or both replicates. Of these, 17.3% tested positive on only the second replicate, indicating that a non-replicate testing method would have missed a substantial fraction of positive cases.

We thank the reviewer for the comments and review of our work. We have provided individual responses to each of the comments bellow.

Major concerns:
• More information is needed on sample selection within the Materials and Methods. Were these swabs collected from symptomatic, suspected positive cases, routine screening of individuals seeking medical care for all maladies, asymptomatic surveillance, or some other reason? This is important for understanding the relevance of the work to pandemic management.

As described in the materials and methods section the nasopharyngeal swabs were received from the Dartford and Gravesham NHS Trust, the Medway NHS Foundation Trust and the Oxleas NHS Foundation Trust (hospital admissions, A&E, elective surgeries, cancer patients, staff), as well as Dartford and Medway care home residents.

The nasopharyngeal swab collection represents testing of general public individuals, who were admitted to hospital or attended A&E for COVID-19 symptoms or other health reasons, e.g., elective surgeries, cancer patients. In addition, nasopharyngeal swabs were collected from care home residents (normal care routine) and healthcare staff (normal work-related testing). Overall, the collection process of nasopharyngeal swabs did not discriminate between symptomatic or asymptomatic individuals and no specific population or patient group was selected for testing for this study. Therefore, we believe that the nasopharyngeal swabs tested in this study accurately represents SARS-CoV2 infections spread at the time in the general public. The manuscript has been updated accordingly.

“A total of 10,014 nasopharyngeal swabs were tested using 20,028 duplicate Real-Time PCR reactions. The tested nasopharyngeal swabs were collected between the months of May and July 2020 and the collection approach was not restricted to COVID-19 symptomatic or SARS-CoV2 infected asymptomatic individuals. Therefore, our nasopharyngeal swab collection method accurately represents the public infection rates at the time of sampling”.

• It is not clear from the methods section and the Ethics Statement that this work has undergone appropriate institutional ethics review. The manner in which patient samples were obtained, the sample identities anonymised, and sample identities tracked and recorded through the replicate testing process was not indicated. If this is a chart review, this should be stated and the appropriate research ethics approvals or exemptions cited. The authors should provide confirmation of ethical review or exemption in accordance with journal and institutional standards: "Any research involving personal information, whether identifiable or anonymised, must be approved via full or proportionate review by an appropriate Research Ethics Committee." Section 7.6.1: <https://www.canterbury.ac.uk/asset-library/research/Governance-and-Ethics/code-of-conduct.pdf>

We apologise for this omission and have made the point clear in the manuscript. This was also clarified with the PLOS One journal at the time of submission. For this study the SARS-CoV-2 testing outcome analysed was carried out as part of the North Kent Pathology Service routine testing workload. We did not recruit patients specifically for this study. Each nasopharyngeal swab following sampling was fully anonymised and was identifiable by a unique number. Research staff that participated in this study were not given access to patient identification numbers and were blinded to any personal or clinical data.

This is a study of testing methodology only and the outcome of SARS-CoV2 RT-PCR testing was analysed with no link to patient clinical information. Therefore, we believe that the study is exempt from ethical review and as such we did not seek ethical approval.

The manuscript has been updated accordingly.

“**Ethical approval**

Ethical approval is not applicable for this study. Patients were not specifically recruited for this study. This is a study of testing methodology only and the outcome of SARS-CoV2 RT-PCR testing was analysed with no link to patient clinical information”.

• Two different testing platforms were used, with different rates of positivity and criteria for assigning a sample as ‘positive,’ yet the data were aggregated. Authors should explain why data aggregation was appropriate in this case, or they should separate the data according to testing platform.

We felt that it is appropriate to aggregate the RT-PCR data in this study and have presented the data as both individual platform and final overall aggregate. Presenting an overall aggregate reflects the reality of testing during the early pandemic stages, taking into account reagent and resources availability, and would align with the systematic early review in regard to false negative results which did not account for platform variance (Arevalo-Rodriguez et al 2020) coupled with the mathematical modelling which also did not address platform variances and assumed 100% accuracy of molecular testing (Grassly *et al* 2020).

As described in the answer to next comment and also in the updated manuscript the same positivity criteria and cutoff values were employed for both testing platforms.

• A major weakness in the interpretation of the results stems from the binary nature of the report of ‘positive’ or ‘negative.’ Depending on how a Cq value cutoff was applied, replicate tests falling near the Cq threshold may be separately categorized as positive or negative, despite experimental results that are almost identical. The authors should examine and/or discuss the degree to which replicate agreement is affected by the application of a Cq cutoff.

Our study followed the published NHS England document: “Guidance and standard operating procedure. COVID-19 virus testing in NHS laboratories” (16^th^ March 2020) on Cq value detection. In addition, our study followed the assay manufacturers’ protocols on Cq value detection.

Overall, samples showing a Cq < 40 and a clear amplification curve, were recorded as positive. Every sample testing reaction included the addition of an internal control to validate the quality and accuracy of the RT-PRC reaction. Therefore, the presentation of our findings is a true reflection of NHS England and PHE guidelines, which informed all UK laboratories undertaking SARS-CoV2 RT-PCR testing early in the pandemic. Since the same Cq cutoff was applied for all reactions, we do not believe that it influences our single vs duplicate testing conclusion.

The manuscript has been updated accordingly.

“One-Step Real-Time RT-PCR reactions were prepared in accordance with the manufacturers’ protocols. Internal controls were included in all RT-PCR reactions, in accordance with the manufacturers’ protocols in order to confirm the accuracy of each RT-PRC reaction”.

“In order to evaluate the effectiveness of a SARS-CoV-2 testing protocol that minimizes the frequency of false negative test results we employed a duplicate PCR reaction approach per tested nasopharyngeal swab. All RT-PCR reactions that resulted in a Ct value lower than 40 cycles and a clear amplification curve, were recorded as positive”.

“In this study we examined whether replicate RT-PCR testing of SARS-CoV-2 RNA, isolated from nasopharyngeal swabs, increases the reliability of testing results’ reporting. We tested 10,014 nasopharyngeal swabs, which equates to 20,028 duplicate Real-Time PCR reactions and identified 491 SARS-CoV-2 positive patients. As mentioned above, RT-PCR reactions were recorded as positive when a Ct value lower than 40 cycles was detected and a clear amplification curve was confirmed. This applies to single and duplicate RT-PCR reactions”.

• Authors have indicated that data are freely available but have not indicated the location or means of obtaining the data.

We apologise for this omission. The data is indeed available upon request and the manuscript has been updated accordingly, with easily identified contact information.

“All quantitative PCR reactions were carried out on the Biorad CFX96 Real-Time PCR detection system. The RT-PCR data is available as a supplementary document”.

Minor concerns:
• The duration and method of RNA storage between swab acquisition and testing, and between testing replicates should be described.

The nasopharyngeal swab collection and sample storage was carried out according to the SOP of the North Kent Pathology Service and we did not seek to interfere with the procedure in order to have an accurate representation of the testing processes that were employed during the early stages of the pandemic. Briefly, the nasopharyngeal swabs were placed in sterile transport media tubes and were kept chilled following collection, during transport and prior to viral RNA extraction. The swabs were processed within 24 hours from collection. The viral RNA (duplicate testing) was tested immediately after extraction. The Methods section in the manuscript has been amended accordingly.

“**Nasopharyngeal swab collection**

Nasopharyngeal swabs were tested at the North Kent Pathology Service (NKPS), Dartford and Gravesham NHS Trust. These were received form the Dartford and Gravesham NHS Trust, the Medway NHS Foundation Trust and the Oxleas NHS Foundation Trust (hospital admissions, A&E, elective surgeries, cancer patients, staff), as well as Dartford and Medway care home residents. Upon sampling each nasopharyngeal swab was placed in a sterile transport media tube and was kept chilled during transport and prior to viral RNA extraction. The nasopharyngeal swabs were processed within 24 hours from collection.

**SARS-CoV-2 RNA extraction**

Total RNA was extracted using HigherPurity™ Viral RNA extraction kit (Cat. No: AN0805-XL) from Canvax Biotech in accordance with the manufacturers protocol. Total RNA was eluted into 40μl of RNAse free water. Total RNA (duplicate testing) was tested immediately after extraction”.

• It appears from the methods that replicate tests were performed on the same platform as the initial test, but this should be explicitly stated.

The manuscript has been updated accordingly.

“All quantitative PCR reactions (single and duplicate testing) were carried out on the Biorad CFX96 Real-Time PCR detection system”.

• In the end of the results section, the authors write, “Testing of 10,014 nasopharyngeal patient swabs has shown that 61.5% of SARS-CoV-2 positive patients were detected on double replicate positive PCR reactions, while approximately 19% of patients were detected as SARS-CoV-2 positive by only one out of the two PCR reaction replicates.” This statement appears to be made in error, since the two values do not add up to 100%. Authors should revisit the intent of this sentence and edit accordingly.

We apologise for the confusion. Our study showed that of all the nasopharyngeal swabs tested with both assay kits, 61.5% were detected as positive on both duplicate RT-PCR reactions with the remainder swabs being detected as positive on one RT-PCR reaction. Of that 17.3% were detected positive on replicate 1 and 21.2% were detected positive on replicate 2. Therefore, we can conclude that approximately 19% of positive nasopharyngeal swabs would be missed if single RT-PCR reactions were used for SARS-CoV2 detection.

The sentence has been edited in the manuscript.

“Testing of 10,014 nasopharyngeal patient swabs has shown that 61.5% of SARS-CoV-2 positive patients were detected on double replicate positive PCR reactions, while 17.3% were detected positive on replicate 1 and 21.2% were detected positive on replicate 2. Overall, we can conclude that approximately 19% of patients were detected as SARS-CoV-2 positive by only one out of the two PCR reaction replicates and these would be missed if single reaction testing was employed”.

• The word ‘outbreaks’ is misspelled in the abstract.
• “corona virus” should be a single word in the Introduction.

The manuscript has been updated accordingly.
